# Improved Quantification by Nuclear Magnetic Resonance Spectroscopy of the Fatty Acid Ester Composition of Extra Virgin Olive Oils

**DOI:** 10.1021/acsfoodscitech.2c00057

**Published:** 2022-07-11

**Authors:** Gabriel Rossetto, Peter Kiraly, Laura Castañar, Gareth A. Morris, Mathias Nilsson

**Affiliations:** Department of Chemistry, University of Manchester, Oxford Road, ManchesterM13 9PL, UK

**Keywords:** NMR, EVOO, DISPEL

## Abstract

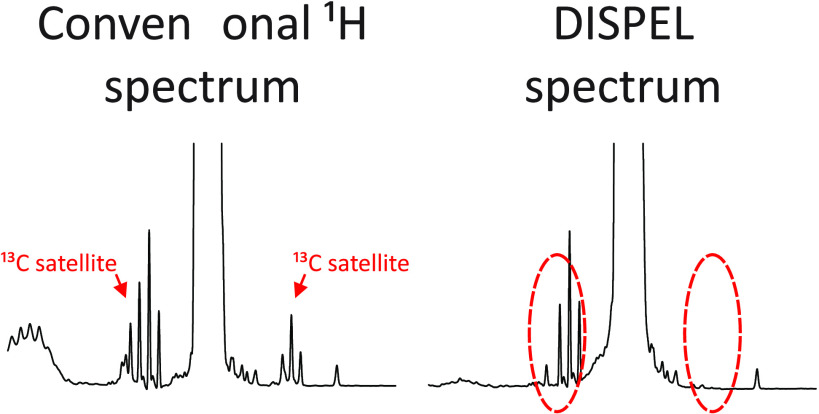

Analysis of foods, which are typically highly complex mixtures, by ^1^H NMR can be difficult because the prevalence of signal overlap complicates characterization and quantification. The various components of a food sample may have a wide range of concentrations, leading to a high dynamic range NMR spectrum and complicating the analysis of less concentrated species. One source of this complication is the presence of ^13^C satellites, peaks that appear either side of a parent peak with ∼0.56% of its intensity. Satellites of concentrated species can easily be comparable in intensity to the signals of minor components, and can partly or wholly obscure them. This is commonly seen in olive oil samples, leading to inaccurate calculation of the fatty acid ester composition of the oil, used for determining the quality of edible oils and for detecting adulteration. Here, we show that the recently introduced Destruction of Interfering Satellites by Perfect Echo Low-pass filtration (DISPEL) experiment is able to suppress ^13^C satellites and can substantially improve the accuracy of integration of minor signals. The DISPEL experiment does not require any complicated optimization, working “out of the box” with standard parameters, and incurs no significant loss of sensitivity. It has the potential to become the default experiment, replacing conventional 1D ^1^H NMR, for quantitative analysis of olive oil.

## Introduction

Nuclear magnetic resonance (NMR) is an attractive technique for the analysis and characterization of mixtures in food research due to its ability both to provide structural information at the atomic level and to quantify individual components. With minimal sample preparation and modest experiment times, NMR is a powerful alternative to the chromatographic techniques that are often used in the field of food analysis. Chromatography requires time-consuming optimization and extraction of specific classes of compounds for the accurate analysis of complex mixtures. Examples of this are seen in the study of phenols in olives using high-performance liquid chromatography (HPLC)^1^ and in the study of plant sterols in foods and vegetable oils using multiple chromatographic techniques such as column chromatography (CC), thin layer chromatography (TLC), and gas chromatography (GC).^2^ Even when coupled with mass spectrometric detection, it is not straightforward to identify the separated compounds, as seen in a study of mycotoxins in food and feed using hyphenated chromatographic techniques.^3^ Furthermore, full quantification is time-consuming, making chromatography unsuitable for the screening of complex mixtures on a large scale. Quantification of oleocanthal (a natural phenolic compound) in olive oil extracts, for example, has proven to be difficult using HPLC as the oleocanthal can react spontaneously with mobile phases such as water or ethanol.^4^ The presence of phenols in extra virgin olive oils (EVOOs) has attracted considerable attention over the years since studies have shown that the phenolic and polyphenolic contents of EVOOs are linked to a lowering of the risk of diseases and cancers. This is attributed to their antioxidant and anti-inflammatory activity;^5^ each phenolic compound has a different antioxidant capacity and health benefits.^6^ In the case of oleocanthal, NMR proved to be superior to HPLC due to its non-invasive nature, not altering the sample under investigation, but is much less sensitive than chromatographic methods. An extensive study on the phenolic fraction of EVOOs, combining liquid chromatography techniques and NMR, provided a good comparison between the techniques.^7^

The simplest NMR experiments, which require only a single radiofrequency (RF) pulse followed by acquisition of the NMR signal (e.g., the conventional 1D ^1^H pulse-acquire experiment), typically take only 5–15 s. In principle, they can provide the user with enough data to characterize a sample based on the chemical shifts (δ), signal integrals, scalar coupling constants (*J*), and multiplet structures. A satisfactory analysis of a simple mixture (structure elucidation and/or characterization) is sometimes possible using only the information provided by a basic 1D ^1^H spectrum, particularly if there is little spectral overlap. However, this is rarely the case for samples with complex spectra such as those of EVOOs, which are the focus of this study. Although a 1D spectrum can be sufficient for basic quantification, as shown below, more sophisticated experiments such as multidimensional NMR are often needed for structure determination.

Integrals of NMR signals can be used straightforwardly for quantitative analysis because the integral of a given signal is directly proportional to the number of nuclei responsible for it. This property has been extensively used in food analysis by NMR, for example, to quantify polysaccharides in food products^8^ and organic compounds in thin stillage^9^ and to determine the fatty acid composition of pork meat.^10^ For the accurate quantification of low concentration components in a sample, the 5–15 s experiment times mentioned earlier will often not be sufficient because time averaging is needed to ensure a high enough signal-to-noise ratio (SNR).

When analyzing complex mixtures with NMR, two problems tend to dominate: sensitivity and spectral resolution. While there have been considerable improvements in sensitivity, with the advent of cryoprobes^11^ and other technological advances, analyzing minor components in complex mixtures is still not straightforward, especially in high dynamic range mixtures. The problem of spectral resolution derives from the overlapping of signals. In complex mixtures, there are many components and hence many signals. In EVOOs, for example, these components belong to multiple representatives of related classes of organic molecules (e.g., free fatty acids and triglycerides), meaning that there are often many similar and closely related signals in a small chemical shift range. Since most signals in ^1^H NMR are also multiplets, analysis becomes challenging as neither the individual signals nor their multiplet structures can be directly identified. Pure shift NMR methods can help by collapsing multiplet structures, giving only a singlet signal for each distinct chemical shift, but this complicates quantitative analysis.^12^

EVOO is extracted by purely mechanical processes from the fruit of *Olea europaea* L., so the glyceric structure of the oil is preserved. It is a staple ingredient around the world, especially in the Mediterranean diet, where it is the principal source of dietary fatty acids (FAs). EVOOs consist primarily (∼98%) of triacylglycerols and secondarily of minor components such as free fatty acids, mono- and diacylglycerols, lipids, and a wide range of phenolic compounds. Cis-mono- and polyunsaturated FAs are known to have positive implications for health, while saturated and trans FAs have negative health implications.^13^ For this reason, the characteristic fatty acid ester composition (FEC) of an EVOO (determined by its percentage makeup of saturated fatty ester (SFE), cis-monounsaturated fatty ester (MUFE), and polyunsaturated fatty ester (PUFE)) is mandatory for its nutritional labeling, as declared by Regulation 1169/2011 of the European Union.^14^ Authentication and quality assessment of EVOOs have also been major issues due to the economic and health implications of fraudulent labeling of olive oils.^15^ The FECs of vegetable oils have been shown by statistical methods to be influenced by cultivar,^16^ geographical origin,^17^ and harvest date.^18^

**Figure 1:**
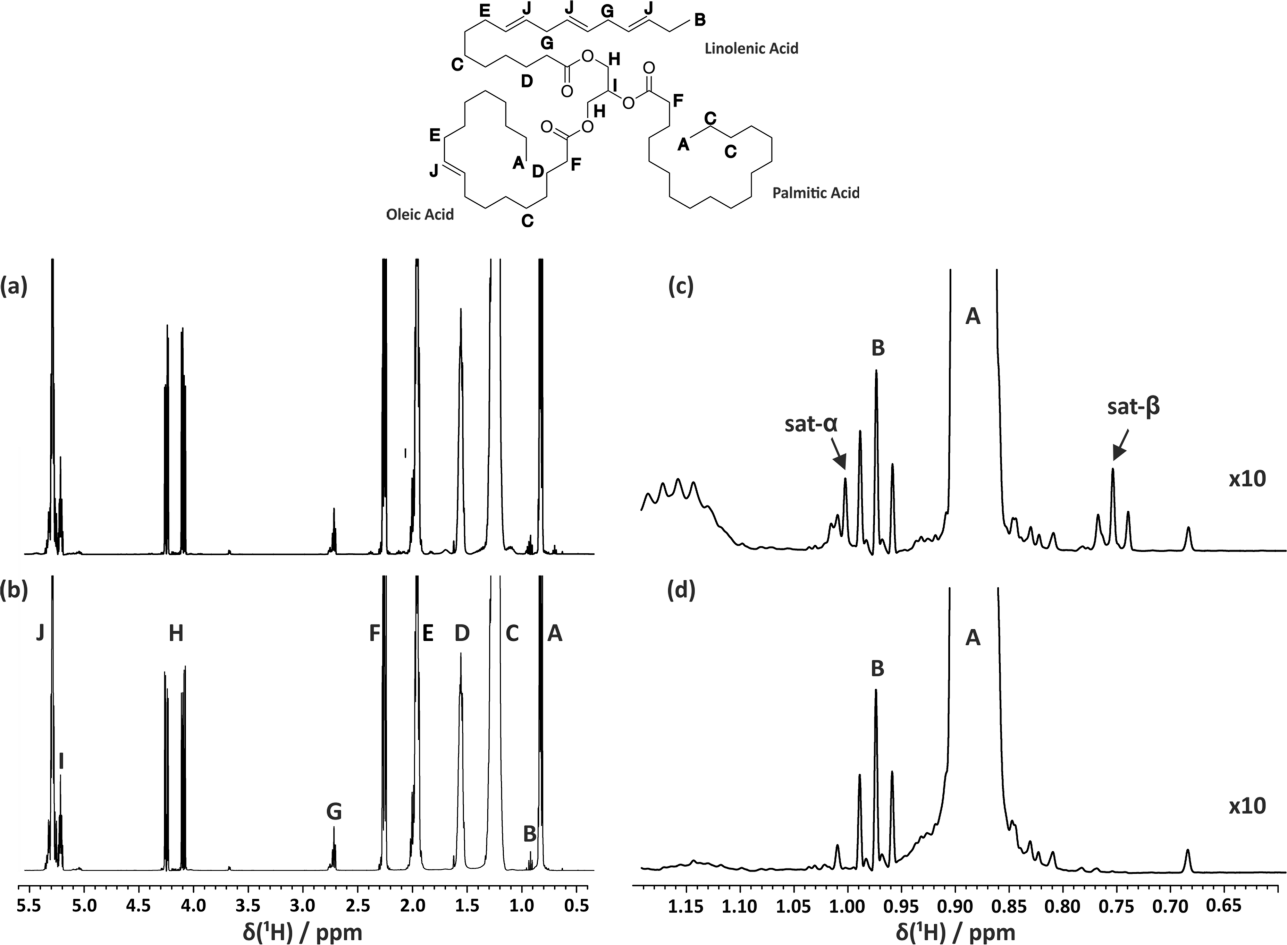
500 MHz (a) conventional ^1^H NMR and (b) DISPEL spectra for sample EVOO1 with signals assigned to a glycerol unit and the fatty acid chains of palmitic, oleic, and linolenic acids. Panels (c) and (d) are vertical expansions of panels (a) and (b), respectively, showing the spectral region around **A** and **B**. The overlap of the ^13^C satellite of **A** with **B** is seen in panel (c), with the removal of the ^13^C satellites by DISPEL in panel (d), which allows for signal **B** to be more accurately integrated.

The attraction of the simple 1D ^1^H pulse-acquire experiment is that it is a fast, efficient, and sensitive (by the standards of NMR experiments) method for investigating an EVOO sample, for example, in determining the FECs of EVOOs.^19,20^ However, even at resonance frequencies of 500 MHz or higher, resolution remains a problem, as can be seen in Figure 1a,c, where the ^13^C satellite of **A** (methyl group from the fatty acid chains, excluding α-linolenic acid) overlaps with **B** (methyl group from α-linolenic acid). ^13^C satellites appear either side of their parent peak, with ∼0.56% of its intensity. They are caused by scalar coupling between the ^1^H and ^13^C nuclei, which have a natural abundance of ∼1.11%. In Figure 1c, the coupling responsible for the spectral overlap is the one-bond coupling (^1^*J*_CH_) of methyl protons **A** with the methyl carbon. Since the satellite of **A** is comparable in intensity to signal **B**, accurate integration of **B** becomes difficult.^19^ This is problematic as the integral of **B** is used in calculating the FEC of an EVOO.^19−21^ One study circumvented this issue by choosing a lower magnetic field of 300 MHz, sacrificing sensitivity and resolution to shift the satellite and signal **B** away from each other.^20^ This solution is not as simple as it seems, however, since different overlaps between satellites and signals of interest will in general require different magnetic field strengths (if indeed such a field exists) to lift the degeneracy between the signal and satellite. Multiple-bond couplings between ^1^H and ^13^C also cause satellite peaks, but because of their much smaller magnitude, they tend to be buried in the base of the parent proton multiplet and hence are included when the multiplet is integrated.

Here, we demonstrate the use of a modification to the 1D ^1^H pulse-acquire experiment that is designed to remove one-bond ^13^C satellite signals. Applying this DISPEL (Destruction of Interfering Satellites by Perfect Echo Low-pass filtration) method^22^ (see Figure S1 for pulse sequence) to EVOOs allows their FECs to be determined more accurately. The cost in sensitivity is negligible, and the experiment does not significantly affect the relative integrals of signals in the spectrum. Five EVOO samples were used in this study to demonstrate the effectiveness of the DISPEL experiment.

## Materials and Methods

All data were acquired, non-spinning, at 298 K on a 500 MHz Bruker Avance NEO spectrometer using a 5 mm room temperature PA TBI 500S2 H/F-BB probe. All chemicals were purchased from Sigma-Aldrich, and the EVOOs were obtained from local supermarkets: EVOO1, EVOO4, and EVOO5 originate from Greece, EVOO2 originates from Spain, and EVOO3 is a mixture of oils from Greece, Italy, Portugal, Spain, and Tunisia. The five samples EVOO1 to EVOO5 were prepared by dissolving 150 μL of the oil in 700 μL of CDCl_3_, with a trace of TMS added as a chemical shift reference. The ^1^H NMR and DISPEL spectra of Figure 1 were acquired with a relaxation delay (d1) of 12 s, four dummy scans, 128 transients, a 10 kHz spectral width with 128 k complex points, and calibrated 90° pulse durations of 12.3 μs at 8.9 W and 17 μs at 147 W for the ^1^H and ^13^C pulses, respectively. All spectra were processed using zero-filling to 262,144 complex points, with reference deconvolution using the TMS signal with a target lineshape of −0.5 Hz Lorentzian component and 1.5 Hz Gaussian. All spectra were manually phased and baseline-corrected in VnmrJ 2.2C.

The DISPEL pulse sequence used here and shown in Figure S1 in the Supporting Information differs slightly from that originally published^22^ because of the need to avoid differential weighting of signals. All gradient pulses and the optional zero-quantum filter were omitted because they can cause diffusion and relaxation weighting, respectively. The full phase cycle of 32 transients obviates the need for gradient pulses and provides the signal-to-noise (SNR) needed for accurate quantification of the smallest integral of interest (SNR of signal **B** > = 2500:1). Zero-quantum suppression^23^ was not needed since clean and in-phase multiplets were observed. If zero quantum suppression were needed in other applications, then either its duration would need to be kept as short as possible or integrals would have to be corrected using *T*_1_s determined experimentally, e.g., by inversion recovery.

## Results

Figure 1a and Figure 1b show the relevant parts of ^1^H and DISPEL spectra, respectively, for sample EVOO1. The high dynamic range of the EVOO spectrum can be appreciated from the very different intensities of signals such as **A** and **B** and the minor signals seen in Figure S3c,d in the Supporting Information. Figure 1c shows the overlap between signal **B** and a ^13^C satellite of signal **A** (labeled sat-α) at 500 MHz. It is clear that it is not possible to choose an integration region for **B** that does not contain some signal from this satellite. After satellite suppression by DISPEL, Figure 1d shows the improvement in the signal shape of the **B** triplet, to give the expected 1:2:1 intensity ratios. As expected, there is a systematic reduction in integral **B** when DISPEL is used, as a result of the suppression of the overlapping ^13^C satellite signal. As mentioned earlier, DISPEL causes negligible loss in sensitivity here compared with the ^1^H pulse-acquire experiment and more importantly has very little effect on the relative integrals of signals in the absence of satellite overlap. Comparing the integrals for **C** in the final two columns of Table 1, the differences are all well below 0.2%.

**Table 1:** Comparison of Relative Integrals of the EVOO Samples for the Standard ^1^H and DISPEL Experiments^b^

EVOO	signal	δ (ppm)	integral region (ppm)	relative integral (^1^H)	relative integral (DISPEL)
	**A**	0.88	0.820–0.948	8.63	8.70
	**B**	0.97	0.961–1.030	0.12	0.11
	**C**	1.30	1.060–1.459	59.12	59.09
	**D**	1.61	1.553–1.705	6.05	5.90
	**E**	2.03	1.948–2.121	9.75	9.79
1	**F**	2.31	2.240–2.382	5.78	5.83
	**G**	2.77	2.675–2.890	0.51	0.51
	**H**^a^	4.22	4.070–4.373	3.85	3.73
	**I**	5.26	5.230–5.299	0.93	0.99
	**J**	5.33	5.300–5.422	5.26	5.35
	**A**	0.88	0.820–0.948	8.74	8.59
	**B**	0.97	0.961–1.030	0.16	0.09
	**C**	1.30	1.060–1.459	58.96	58.92
	**D**	1.61	1.553–1.705	6.03	6.07
	**E**	2.03	1.948–2.121	9.64	9.71
2	**F**	2.31	2.240–2.382	5.75	5.78
	**G**	2.77	2.675–2.890	0.59	0.62
	**H**^a^	4.22	4.070–4.373	3.88	3.87
	**I**	5.26	5.230–5.299	0.97	1.03
	**J**	5.33	5.300–5.422	5.28	5.32
	**A**	0.88	0.820–0.948	8.68	8.67
	**B**	0.97	0.961–1.030	0.13	0.09
	**C**	1.30	1.060–1.459	58.97	58.99
	**D**	1.61	1.553–1.705	6.11	6.00
	**E**	2.03	1.948–2.121	9.76	9.80
3	**F**	2.31	2.240–2.382	5.77	5.83
	**G**	2.77	2.675–2.890	0.52	0.52
	**H**^a^	4.22	4.070–4.373	3.86	3.77
	**I**	5.26	5.230–5.299	0.94	1.00
	**J**	5.33	5.300–5.422	5.26	5.32
	**A**	0.88	0.820–0.948	8.66	8.59
	**B**	0.97	0.961–1.030	0.15	0.08
	**C**	1.30	1.060–1.459	58.98	59.06
	**D**	1.61	1.553–1.705	6.15	6.04
	**E**	2.03	1.948–2.121	9.72	9.72
4	**F**	2.31	2.240–2.382	5.79	5.84
	**G**	2.77	2.675–2.890	0.52	0.53
	**H**^a^	4.22	4.070–4.373	3.88	3.87
	**I**	5.26	5.230–5.299	0.95	0.99
	**J**	5.33	5.300–5.422	5.22	5.29
	**A**	0.88	0.820–0.948	8.69	8.73
	**B**	0.97	0.961–1.030	0.14	0.12
	**C**	1.30	1.060–1.459	59.18	59.14
	**D**	1.61	1.553–1.705	6.03	5.87
	**E**	2.03	1.948–2.121	9.63	9.78
5	**F**	2.31	2.240–2.382	5.77	5.81
	**G**	2.77	2.675–2.890	0.48	0.50
	**H**^a^	4.22	4.070–4.373	3.92	3.72
	**I**	5.26	5.230–5.299	0.95	0.98
	**J**	5.33	5.300–5.422	5.21	5.36
					

a The strongly coupled methylene multiplet **H** from *sn*-1,3 of the triglyceride moiety is distorted by the perfect echo element in the DISPEL experiment, reducing the integral slightly, but this does not affect the FEC calculation used.

b The sums of the relative integrals in each spectrum are normalized to 100.

It should be noted that the form of the methylene multiplets *sn*-1,3 (**H**) of the triglyceride moiety, which are not used in the calculations below, is affected by the DISPEL experiment, as seen in Figure S4, reducing the accuracy of this integral slightly. The peak intensities of multiplet **H** in both the conventional and the DISPEL spectrum deviate from the 1:1:1:1 ratios expected for weak coupling, but the intensity distortions are reversed with DISPEL, as a result of the perfect echo element used in the pulse sequence,^22,24^ very slightly reducing the **H** signal integrals obtained with DISPEL. Since the **H** integrals are not used in the FEC calculations, this effect is not a concern here. With other samples, or at lower magnetic fields where strong coupling could be more problematic, the addition of a final orthogonal π/2 pulse to the perfect echo can be used to suppress the signal intensity distortion, but this will not restore the full integral.^24^

The EVOO samples used in this study all show satellite overlap between signals **I** and **J**, i.e., the one-bond ^13^C satellite from **I** overlaps with **J** and *vice versa*. Because of the complexity of EVOO spectra, it is difficult to show that both **I** and **J** lose an integral value equal to the satellites they overlap with when using DISPEL, but this can be demonstrated using the model AMX system of 2-bromothiophene, as shown in the Supporting Information. The ^13^C satellites of the three CH signals of 2-bromothiophene are seen in Figure S5b to be comparable in intensity to the impurities in the sample, with overlap at ∼7.09 ppm between an unknown impurity (UI) and the ^13^C satellite from signal 4 (S4′). Figure S5c shows the suppression of the satellites using DISPEL, which also removes the overlap between satellite and impurity. The relative integrals of the AMX protons, their satellites, and the overlapping region, for both the conventional ^1^H and DISPEL experiments, are shown in Table S2, where the relative integral of the overlapping region is shown to decrease almost exactly by the integral of S4 in the DISPEL experiment. The AMX sample can also be used to demonstrate the superiority of DISPEL as a spectral editing technique over conventional ^13^C decoupling. Figure S5d shows good suppression of the satellites using ^13^C decoupling during acquisition, but there is severe loss in resolution in the decoupled spectrum because of the short acquisition time of 0.1 s used to avoid excessive sample heating when applying composite pulse decoupling.

The integrals measured for the EVOO samples can be used to calculate their FECs. Using eqs 1–5, from a previous study,^25^ the proportions of saturated fatty ester (SFE), mono-unsaturated fatty ester (MUFE), di-unsaturated fatty ester (DUFE), and tri-unsaturated fatty ester (TUFE) can be calculated from the experimental integrals for the different regions using the following equations:
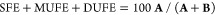
1
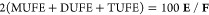
2
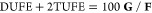
3
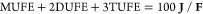
4
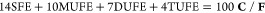
5
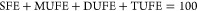
6where the boldface letters correspond to the integrals of the signals shown in Figure 1b.

For the EVOOs studied here, Table 2 shows the FEC values determined with data from ^1^H pulse-acquire and DISPEL experiments using eqs 1–6. Since the system of six equations and four unknowns (SFE, MUFE, DUFE, and TUFE) is overdetermined for three of the unknowns, FEC values were obtained using Mathematica to determine the five compositions that minimize the sum of the squares of the differences between the experimental integral ratios (1–5) and the integral ratios for a given composition. As can be seen from the residuals in Table S3 in the Supporting Information, excellent agreement was obtained in every case. Table 2 shows that, as expected from the known partial overlap between peak **B** and a ^13^C satellite of peak **A**, the conventional method overestimates the percentage of tri-unsaturated ester chains*.* The results of the DISPEL experiments are also of interest in revealing the signals of very low-level components of EVOO whose signals are completely swamped by the ^13^C satellites of the signals of abundant components; several such signals are shown for sample EVOO1 in Figure S3 of the Supporting Information.

**Table 2:** Percentage FECs Obtained for Five EVOO Samples from Both the ^1^H and DISPEL Experiments

EVOO	1		2		3		4		5	
experiment	^1^H	DISPEL	^1^H	DISPEL	^1^H	DISPEL	^1^H	DISPEL	^1^H	DISPEL
SFE	14.5	14.1	15.4	14.9	14.3	13.9	14.5	14.4	15.3	14.2
MUFE	76.0	74.2	74.9	73.7	76.2	74.0	75.4	72.5	75.5	75.0
DUFE	8.2	10.5	7.9	10.3	8.1	11.1	8.5	12.1	7.6	9.5
TUFE	1.4	1.2	1.8	1.1	1.5	1.1	1.7	1.0	1.6	1.3

## Conclusions

The DISPEL experiment has the potential to be a useful new tool in the analysis of food products and is shown here to be highly effective in suppressing interfering ^13^C satellites in the ^1^H NMR spectrum of five EVOO samples. With negligible loss in signal intensity and no difference in resolution compared with the standard 1D ^1^H NMR experiment, it allows straightforward qualitative and quantitative analysis of the components of high dynamic range mixtures.

## Supporting Information Available

The DISPEL pulse sequence, a further use of the DISPEL experiment for analysis of the minor components in EVOOs whose signals would otherwise be hidden under the more intense ^13^C satellites in the spectrum, data from experiments on a model AMX spin system, and experimental data and residuals for the fitting of experimental EVOO results to eqs 1–6 (PDF)

## Notes

Pulse sequence code and software can be downloaded from our web site (https://nmr.chemistry.manchester.ac.uk/) and raw experimental data from DOI 10.48420/19130753.
