# Supplementary material for: Improved Quantification by Nuclear Magnetic Resonance Spectroscopy of the Fatty Acid Ester Composition of Extra Virgin Olive Oils
Source: ACS Food Sci Technol. 2022 Jul 11;2(8):1237–42. doi: 10.1021/acsfoodscitech.2c00057 (PMC9396653; doi:10.1021/acsfoodscitech.2c00057)
Supplement: Supplementary file 1 — fs2c00057_si_001.pdf [file fs2c00057_si_001.pdf]

Supporting Information

Improved quantification by NMR spectroscopy of the fatty acid composition of extra virgin olive oils

Gabriel Rossetto[a], Peter Kiraly[a,‡], Laura Castañar[a], Gareth A. Morris[a], and Mathias Nilsson[a*]

[a]Department of Chemistry, University of Manchester, Oxford Road, Manchester, M13 9PL, UK
[‡]Present Address: JEOL UK Ltd., Bankside, Long Hanborough, OX29 8SP, UK

*Email: mathias.nilsson@manchester.ac.uk

Table of Contents

Experimental details **3**

Figure S1 – DISPEL pulse sequence 3

Table S1 – phase cycle for DISPEL experiment.. 3

EVOO 1 spectra.. **4**

Figure S2 – ^{1}H spectrum for the EVOO 1 sample 4

Figure S3 – minor components under suppressed satellites 5

Figure S4 – effect of perfect echo element on signal **H** 6

AMX spectra **7**

Figure S5 – suppression of ^{13}C satellites for by DISPEL and ^{13}C decoupling 7

Table S2 – relative integrals of AMX signals and their satellites.. 8

EVOO data fitting **9**

Table S3 – Experimental and back-calculated integral ratios and residuals for data from conventional experiments on the five EVOO samples. 9

Table S4 – Experimental and back-calculated integral ratios and residuals for data from DISPEL experiments on the five EVOO samples. 10

Experimental details

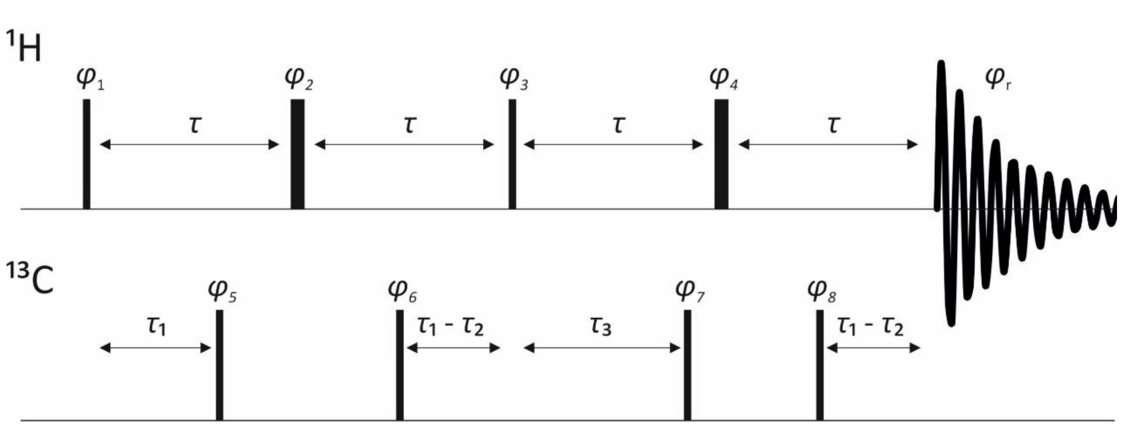

Figure S1. DISPEL pulse sequence used here. The narrow and wide black rectangles represent 90° and 180° pulses, respectively. The τ delay was set to 4.05 ms, and τ_1, τ_2, τ_3 and τ_4 to 3.2, 1.1, 3.95 and 1.56 ms, respectively, giving excellent satellite suppression for all ^1H-^{13}C coupling constants from 120 to 360 Hz.[1]

Table S1. Phase cycle for each pulse and the receiver in the DISPEL experiment.

Pulse	Phase Cycle
Φ_1	x, -x
Φ_2	x_8, y_8, $-x_8$, $-y_8$
Φ_3	y, -y
Φ_4	$-x_2$, $-y_2$, x_2, y_2
Φ_5	x, -x
Φ_6	x_2, $-x_2$
Φ_7	x_4, $-x_4$
Φ_8	x_8, $-x_8$
Φ_r	x, $-x_2$, x_2, $-x_2$, x, -x, x_2, $-x_2$, x_2, -x

EVOO 1 spectra

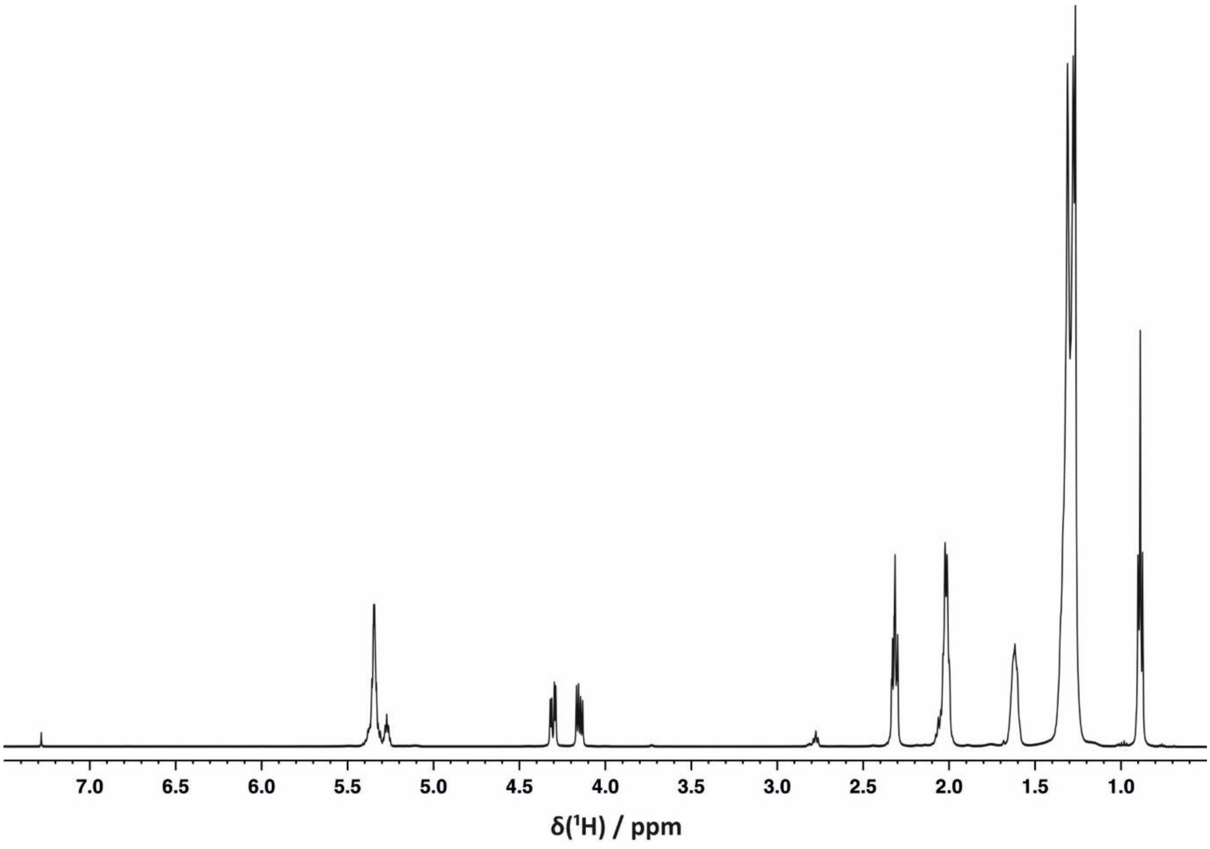

Figure S2. Full ^{1}H NMR spectrum of the EVOO 1 sample.

The two spectral regions displayed in **Figure S3** are regions of the EVOO 1 spectrum where the ^{13}C satellites of **H** and **E/F**, respectively, overlap with signals of minor components marked with asterisks in **Figures S3c** and **d**. These signals are only revealed after satellite suppression by DISPEL, demonstrating how characterisation, or even observation, of these signals would be very difficult with only a ^{1}H pulse-acquire experiment.

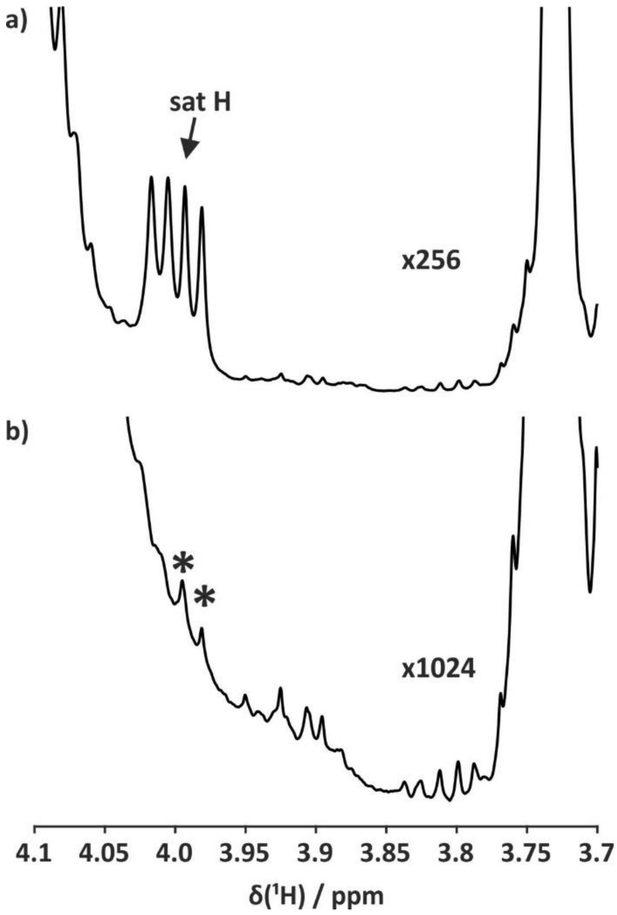

Figure S3. 500 MHz (a, c) conventional ^{1}H NMR and (b, d) DISPEL spectra for the EVOO 1 sample. (a, b) and (c, d) are vertical expansions of **Figures 1a** and **1b,** respectively, showing two spectral regions where minor signals overlap with ^{13}C satellites. The asterisks mark the signals of minor components which are revealed after satellite suppression by DISPEL.

The methylene protons *sn-1,3* of signal **H** are strongly coupled to each other, as can be seen by the "roofing" effect in the ^{1}H spectrum in **Figure S4a**, distorting the multiplets from the 1:1:1:1 relative peak inetnsities expected in the case of weak coupling. When DISPEL is used, the roofing effect is reversed, as shown in **Figure S4b**.

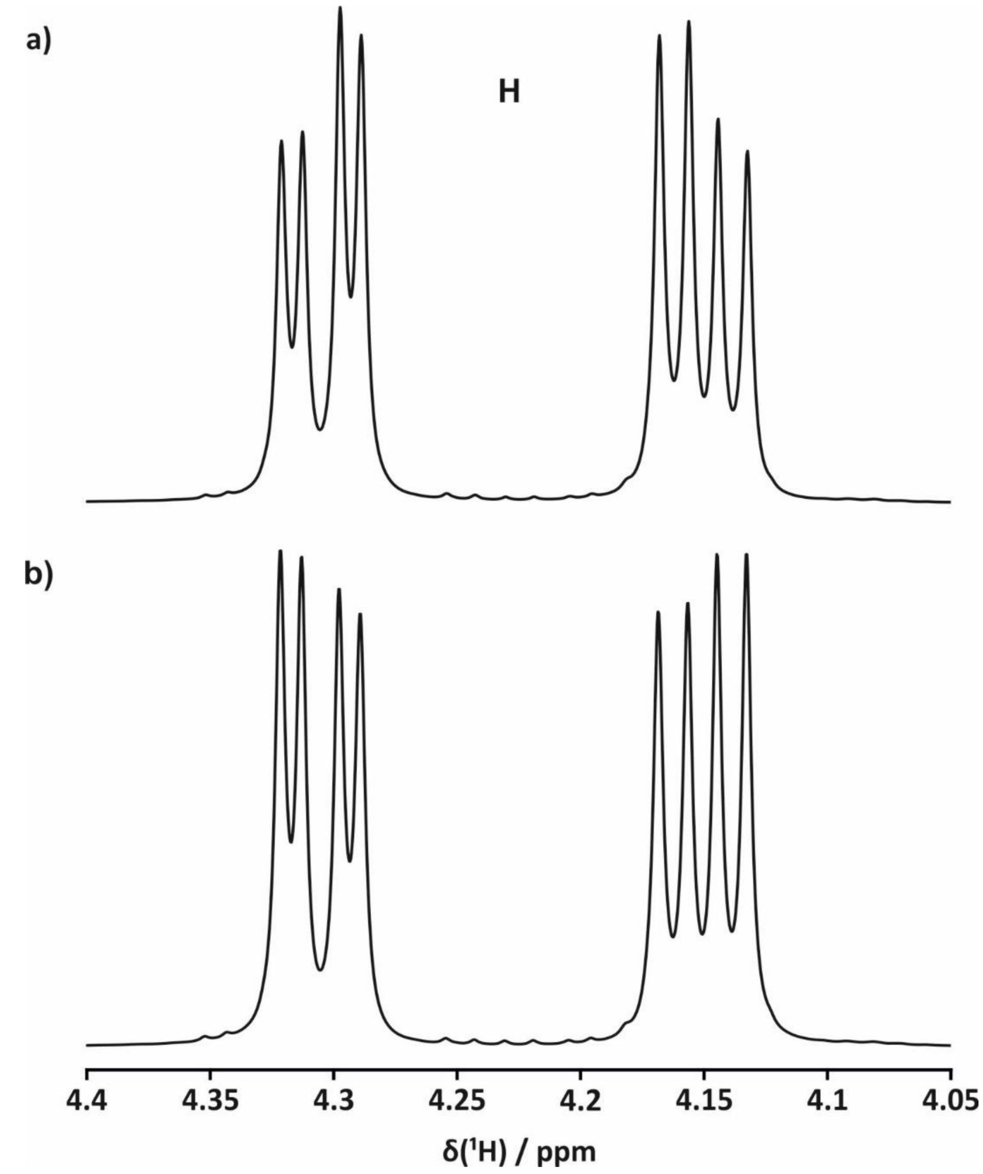

Figure S4. 500 MHz (a) conventional ^{1}H NMR and (b) DISPEL spectra showing the effects of DISPEL on the diastereotopic CH_2s of **H** of the EVOO 1 sample. The roofing in (a) caused by strong coupling between the two protons is reversed by the DISPEL sequence, as shown in (b). Integration of (b) would give a slightly different value, but as **H** is not used for the FEC determination here this is not a problem.

AMX spectra

A sample of 2-bromothiophene (AMX spin system) is used to demostrate the effectiveness of the DISPEL experiment at surpressing ^{13}C satellites and for quantitation purposes. **Figure S5c** shows the DISPEL spectrum of the AMX sample and **Figure S5d** the spectrum with GARP-4 ^{13}C composite pulse decoupling. The superiority of the DISPEL experiment is clear; there is no loss in resolution, whereas the decoupling experiment suffers because of the limited acquisition time usable. Because of the complexity of EVOO spectra it is difficult to show that both **I** and **J** lose an integral value equal to that of the satellites they overlap with when using DISPEL, but the equivalent change can be demonstrated straightforwardly in the simpler spectrrum of 2-bromothiophene, as seen in **Table S2**. The relative integral of of the S4'+UI region decreases in the DISPEL experiment by almost exactly the integral of S4.

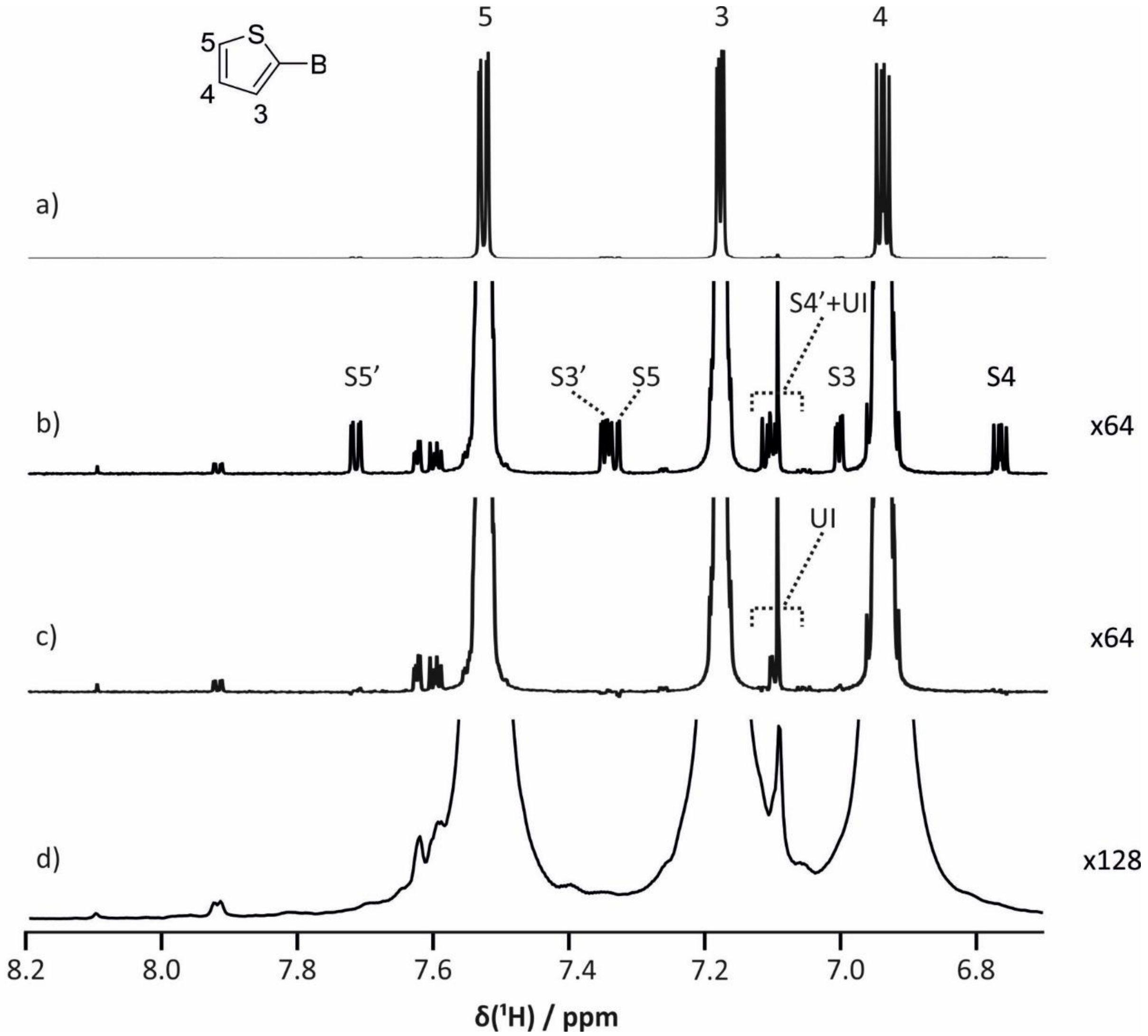

Figure S5. 500 MHz ^{1}H spectra of 2-bromothiophene in DMSO-d_6: (a) conventional spectrum, (b) expansion of conventional spectrum showing ^{13}C satellites and impurities, (c) expansion of DISPEL spectrum showing suppression of ^{13}C satellites along with the removal of the overlap between S4' with an unknown impurity (UI), and (d) conventional spectrum acquired using GARP-4 ^{13}C composite pulse decoupling (0.4 s acquisition time),.

Table S2. Comparison of relative integrals of the 2-bromothiophene sample for the standard ^{1}H and DISPEL experiments. The sum of the integrals was normalised to 100.

signal	δ	integral region / ppm	relative integral (^{1}H)	relative integral (DISPEL)
S5’	7.71	7.733-7.684	0.19	- [a]
5	7.52	7.570-7.460	33.03	33.40
S3’+S5	7.33	7.365-7.297	0.39	- [a]
3	7.17	7.193-7.146	32.70	33.16
S4’+UI	7.09	7.114-7.076	0.48	0.28 [b]
S3	7.00	7.022-6.974	0.26	- [a]
4	6.93	6.965-6.899	32.78	33.09
S4	6.76	6.782-6.736	0.18	- [a]

[a] Signal intensity is too low to integrate accurately.
[b] Only UI as DISPEL removes S4’

EVOO data fitting

FECs of the five EVOO samples were determined by minimising the sum of the squares of the differences between the experimental values of the integral ratios in the right hand sides of equations (1) to (5) and the integral ratios back-calculated from trial compositions. **Tables S3 and S4** show the experimental integral ratios, back-calculated ratios and residuals for data from the conventional ^{1}H spectra and the DISPEL spectra. Only three of the parameters are over-determined (because of the choice in reference 25 to integrate methyl protons spearately from methylene) so the residuals are zero for integral ratio (1).

Table S3. Experimental and back-calculated integral ratios and residuals for data from conventional experiments on the five EVOO samples.

	eq (1)	eq (2)	eq (3)	eq (4)	eq (5)
EVOO sample	Experimental ratios				
1	98.6	168.7	8.8	91.1	1023.1
2	98.2	167.7	10.3	91.9	1025.7
3	98.5	169.1	9.0	91.1	1021.4
4	98.3	167.9	8.9	90.1	1019.1
5	98.4	167.0	8.4	90.4	1026.4
	Back-calculated ratios				
1	98.6	170.9	10.9	96.3	1025.5
2	98.2	169.1	11.4	96.0	1027.5
3	98.5	171.4	11.0	96.8	1024.0
4	98.3	171.0	11.8	97.3	1022.4
5	98.4	169.4	10.8	95.5	1028.8
	Residuals				
1		2.2	2.0	5.2	2.4
2		1.4	1.1	4.1	1.7
3		2.3	2.1	5.7	2.6
4		3.2	2.9	7.2	3.4
5		2.4	2.4	5.1	2.5

Table S4. Experimental and back-calculated integral ratios and residuals for data from DISPEL experiments on the five EVOO samples.

	eq (1)	eq (2)	eq (3)	eq (4)	eq (5)
EVOO sample	Experimental ratios				
1	98.8	167.9	8.8	91.8	1014.0
2	98.9	168.1	10.7	92.0	1020.0
3	98.9	168.0	9.0	91.3	1011.9
4	99.0	166.3	9.0	90.5	1010.9
5	98.7	168.2	8.6	92.1	1017.5
	Back-calculated ratios				
1	98.8	171.8	12.9	98.8	1017.7
2	98.9	170.1	12.4	97.5	1022.4
3	98.9	172.2	13.2	99.3	1016.0
4	99.0	171.2	14.0	99.6	1015.6
5	98.7	171.5	12.1	97.9	1020.6
	Residuals				
1		3.9	4.1	7.0	3.7
2		2.0	1.7	5.5	2.4
3		4.2	4.2	8.0	4.1
4		4.9	5.0	9.1	4.7
5		3.3	3.5	5.7	3.1

1. Moutzouri, P.; Kiraly, P.; Phillips, A. R.; Coombes, S. R.; Nilsson, M.; Morris, G. A., 13C Satellite-Free 1H NMR Spectra. *Anal Chem* **2017,** *89* (22), 11898-11901.
